# An investigation of the dual mechanism of uORF-mediated regulation of GGP1 expression and the effects on drought stress in cotton

**DOI:** 10.1093/plphys/kiag693

**Published:** 2026-10-19

**Authors:** Menghan Liu, Chao Li, Juyun Zheng, Yunpeng Cai, Peng Wang, Jiachen Yuan, Ye Wang, Mengyue Yan, Xueyuan Li, Sean Bulley, Xiaoyang Ge, Jingjing Zhan

**Affiliations:** School of Life Sciences, Zhengzhou University, Zhengzhou, Henan 450001, China; State Key Laboratory of Cotton Bio-Breeding and Integrated Utilization, Institute of Cotton Research of Chinese Academy of Agricultural Sciences, Anyang, Henan 455000, China; Xinjiang Key Laboratory of Cotton Genetic Improvement and Intelligent Production, Cotton Research Institute of Xinjiang Academy of Agricultural Sciences, Urumuqi, Xinjiang 830091, China; State Key Laboratory of Cotton Bio-Breeding and Integrated Utilization, Institute of Cotton Research of Chinese Academy of Agricultural Sciences, Anyang, Henan 455000, China; State Key Laboratory of Cotton Bio-Breeding and Integrated Utilization, Institute of Cotton Research of Chinese Academy of Agricultural Sciences, Anyang, Henan 455000, China; State Key Laboratory of Cotton Bio-Breeding and Integrated Utilization, Institute of Cotton Research of Chinese Academy of Agricultural Sciences, Anyang, Henan 455000, China; State Key Laboratory of Cotton Bio-Breeding and Integrated Utilization, Institute of Cotton Research of Chinese Academy of Agricultural Sciences, Anyang, Henan 455000, China; State Key Laboratory of Cotton Bio-Breeding and Integrated Utilization, Institute of Cotton Research of Chinese Academy of Agricultural Sciences, Anyang, Henan 455000, China; Xinjiang Key Laboratory of Cotton Genetic Improvement and Intelligent Production, Cotton Research Institute of Xinjiang Academy of Agricultural Sciences, Urumuqi, Xinjiang 830091, China; New Cultivar Innovation, New Zealand Institute for Bioeconomy Science Limited, Te Puke 3182, New Zealand; School of Life Sciences, Zhengzhou University, Zhengzhou, Henan 450001, China; State Key Laboratory of Cotton Bio-Breeding and Integrated Utilization, Institute of Cotton Research of Chinese Academy of Agricultural Sciences, Anyang, Henan 455000, China; Xinjiang Key Laboratory of Crop Gene Editing and Germplasm Innovation, Institute of Western Agriculture, CAAS, Changji, Xinjiang 831100, China; School of Life Sciences, Zhengzhou University, Zhengzhou, Henan 450001, China; State Key Laboratory of Cotton Bio-Breeding and Integrated Utilization, Institute of Cotton Research of Chinese Academy of Agricultural Sciences, Anyang, Henan 455000, China

## Abstract

Upstream open reading frames (uORFs) are intrinsic regulatory elements that fine-tune gene expression at the translational level without altering genomic sequences or protein structures. Here, we targeted the conserved uORF that resides in the 5'-untranslated region (5'-UTR) of a GDP-galactose phosphorylase gene (*GhGGP1*) in cotton (*Gossypium hirsutum*). Regulation of GGP1 is a key control point of plant L-ascorbic acid biosynthesis. Using a dual-luciferase reporter system, we demonstrate that this uORF represses translation of the downstream GGP1 coding sequence in a feedback manner in response to elevated ascorbate, confirming previous findings in other species. Furthermore, CRISPR/Cas9-mediated disruption of the uORF_GhGGP1_ generated homozygous *uorf_GhGGP1_* mutants with unchanged transcript levels but markedly increased protein abundance and L-ascorbic acid accumulation. Under drought stress, these mutants exhibit reduced malondialdehyde levels, enhanced total antioxidant capacity, and improved drought tolerance. Mechanistically, we further show that under drought conditions, the transcription factor GhBBM directly binds to the uORF-containing cis-regulatory element, enhancing (*GhGGP1*) transcription and drought resistance. Together, our findings establish the uORF_GhGGP1_ as a dual-layer regulatory module that integrates transcriptional activation by GhBBM with translational control of the downstream protein encoding open reading frame, thereby fine-tuning drought stress responses in cotton.

## Introduction

The upstream open reading frame (uORF), located in the 5'-untranslated region (5'UTR) of some *mRNA*s, belongs to a class of *mRNA* elements that can precisely control translation of the downstream protein encoding open reading frame (pORF) (Guo et al. 2022). In contrast to conventional genome-editing approaches that directly target protein-coding sequences, editing upstream open reading frames (uORFs) can offer a different regulatory strategy to that of either disrupting the target enzyme function encoded in the pORF or altering the enzyme activity (increasing or reducing enzyme activity). Editing of the uORF offers the potential to adjust or remove fine-tuning of translational efficiency. This enhances the predictability and safety of trait improvement, avoiding the potentially unforeseen adverse effects or loss of function that may arise from altering protein structure or completely eliminating protein or enzyme function such as the creation of null mutants (Hayden and Jorgensen 2007; Tran et al. 2008; Zhang et al. 2018; Li et al. 2018b). Notably, editing of uORFs allows for the straightforward generation of transgene-free homozygous mutants through segregation in subsequent generations. Essentially, one is creating sometimes novel allelic variation that can be easily incorporated into conventional breeding programs. This characteristic aligns with the regulatory frameworks for genome-edited crops in many countries and regions, facilitating the rapid certification and commercialization of new varieties (Um et al. 2021).

GDP-L-galactose phosphorylase gene (*GGP1*) is also known as *VTC2* (the original Arabidopsis mutant code name) and is the main control point of ascorbate (AsA) biosynthesis (Bulley and Laing 2016; Fenech et al. 2021; Baldet et al. 2024). Its expression is not only regulated transcriptionally but also translationally, because when higher AsA levels are detected (by a currently unknown mechanism), the point of translation initiation on the *GGP1 mRNA* is shifted upstream to the non-canonical start codon of the highly conserved uORF in the 5'UTR rather than the downstream GGP1 enzyme pORF (Laing et al. 2015). The predicted uORF peptide is thought to act by stalling the ribosome (Laing et al. 2015). The translational regulation of pORFs by highly conserved uORFs enables plants to fine-tune their gene expression under different environmental conditions, which has been confirmed in several plant studies (Zhang et al. 2020).

Increased biosynthesis of AsA in model and crop species has demonstrated that even minor increases in AsA can confer enhanced tolerance to a broad range of abiotic stresses (Lisko et al. 2014; Macknight et al. 2017; Xiao et al. 2021). Hence, herein we have used the approach of gene editing to elevate AsA by targeting the uORF of GGP1 in cotton, with a goal of increasing tolerance to abiotic stress in cotton.

Transcription factors are important regulatory proteins in cells, and they play a central role in the regulation of gene expression. Transcription factors regulate the transcriptional activity of genes by binding to specific DNA sequences to either promote or repress transcription and thereby affecting cell fate and organism development. To date, several transcription factors (TFs) have been reported to be involved in regulating AsA biosynthesis (see the review by Castro et al. 2023), with more being discovered reported since. In cotton, relevant studies on transcription factors regulating AsA biosynthetic regulation of *GGP1*, the rate-limiting gene in AsA biosynthesis, remain limited.

Collectively, our study demonstrates the positive role of *GhGGP1* in cotton drought tolerance, which is governed by a 2-tiered regulatory mechanism. We show that the transcription factor GhBBM directly activates *GhGGP1* expression through binding to a cis-element within its uORF. In parallel, we show that CRISPR/Cas9 editing of the uORF_GhGGP1_ alleviates its translational repression, thereby enhancing GGP1 enzyme translation in planta. These findings provide novel insights into leveraging uORFs for developing elite germplasm and offer a theoretical underpinning for breeding drought-tolerant cotton varieties.

## Materials and methods

### Plant materials and treatment

Cotton transformation experiments were performed at the transgenic platform of the State Key Laboratory of Cotton Biology, Chinese Academy of Agricultural Sciences. The uORF mutants *uorf_GhGGP1_*, together with numerous transgenic cotton lines, including *GhBBM*-knockout lines (KO-*GhBBM*) and *GhBBM*-overexpressing lines (OE-*GhBBM*), were all derived from *G. hirsutum* cultivar ZM24 (Ge et al. 2023). Transgenic cotton plants were grown in the cultivation room at a temperature of 25 ± 3 °C, 50% to 75% relative humidity, and a 16/8-h light/dark cycle. Seedlings of *Nicotiana benthamiana* were established and maintained in a controlled greenhouse at 23 ± 2 °C, with a relative humidity of 60% to 70% and white light illumination under long daylight conditions (16/8-h light/dark cycle).

For germination analysis, genetically modified and wild-type cotton seeds were placed in wet gauze supplemented with a solution of 10% PEG6000, and the germination rates were subsequently recorded. For the root length experiments, genetically modified and wild-type cotton seeds were placed in wet gauze to accelerate germination. When radicles emerged, the seeds were subsequently placed upright in germination bags containing a solution of 10% PEG6000. The primary root lengths were measured after 4 d. Each assay consisted of at least 3 independent biological replicates.

### Plasmid construction

All vectors were constructed using the one-step Gateway cloning method that facilitates high-throughput cloning of target sequences (Choudhury et al. 2017). The high-performance TKS Gﬂex DNA polymerase (Takara, Tokyo, Japan) was used to amplify all the target gene fragments by polymerase chain reaction (PCR), and the ClonExpress II One Step Cloning Kit (Vazyme, Nanjing, China) was used to construct the vectors. All primers were designed with the inclusion of a 15 to 21 bp sequence of the vector as the homologous arm and a corresponding double restriction endonuclease site adaptor for the ampliﬁed gene, in addition to a 15 to 30 bp target gene sequence (Table S1). The target gene fragments were ampliﬁed by PCR using either cDNAs or cotton genomic DNA as the template, which were ligated into the vector with the same homologous arm and restriction site.

To perform targeted mutation of *GhGGP1* (GH_D05G3942, GH_D01G0810, GH_A01G0801, GH_D13G1566) in the *G. hirsutum* ZM24 genome, the full complementary DNA sequences of *GhGGP1* were retrieved from the Cotton Functional Genomics Database (CottonFGD), and its uORF–pORF boundaries were determined. Targeted mutagenesis in the uORF region was performed using the CRISPR/Cas9 genome editing system, and the pCBC-DT1T2 and pKSE401 vectors were used to construct the CRISPR-*GhGGP1* vector (Xing et al. 2014). Four speciﬁc sgRNAs, sgRNA-*GhGGP1*-A01(5'-TAGACAAATTGTGGACATCACGG-3'), sgRNA-*GhGGP1*-D13(5'-GTTACACATTGTGGACATCACGG-3'), sgRNA-*GhGGP1*-D01(5'-TAGTCAAATTGTGGACATCACGG-3'), and sgRNA-*GhGGP1*-D05b(5'-TAGACAAATTGTGGGCATCACGG-3'), were designed using the CRISPR REGN tools (CRISPR RGEN Tools). The pCBC-DT1T2 plasmid was used as a template for primer PCR ampliﬁcations. The PCR product was puriﬁed before being cloned into the pKSE401 vector using the enzyme digestion–ligation system (2 μL of PCR product, 2 μL pKSE401 plasmid, 1.5 μL10 × T4 ligase buffer, 1.5 μL 10 × BSA, 1 μL *BsaI*, 1 μL T4 ligase, and 6 μL ddH_2_O), which were incubated at 37 °C for 5 h, followed by 50 °C for 5 min, before being terminated by heat inactivation at 80 °C for 10 min. T4 ligase buffer, T4 DNA ligase, and BsaI were purchased from New England Biolabs (Ipswich, MA). The AT26 and WMC016 vectors were used to construct the KO-*GhBBM* vector. The primers used for the construction of the gene editing vector and those used for identifying the edited sites are listed in Table S1. The potential target-missing rate was evaluated using Cas-Designer in CRISPR RGEN tools (http://www.rgenome.net/).

### Dual-luciferase assay in *N. benthamiana*

For the dual-luciferase (LUC/REN) reporter assay system in tobacco leaves, the pro*GhGGP1*-A01:LUC vector was generated, whereby the *GhGGP1* promoter fragment was cloned into the pGreenII0800 vector with REN as an internal reference, which was pre-digested by *BamHI* and *XhoI* (Edwards et al. 2022). To generate the 35S: *GhBBM* effector plasmids, the CDS of *GhBBM* was cloned into the WMV069 vector that was pre-digested with *BamHI* and *XhoI*. After sequencing, the verified plasmids were transformed into GV3101. The Agrobacterium harboring reporter and effector constructs was co-infiltrated into *N. benthamiana* leaves. The WMV069 empty vector with the reporter was used as a negative control. Luciferase signals were captured and analyzed using a Tanon 5200 Multi Chemiluminescent Imaging System (Tanon, Shanghai, China). Firefly luciferase (LUC) and Renilla luciferase (REN) activity levels were measured using a dual-luciferase reporter assay system (Promega). The measured LUC activity was normalized to that of REN.

### Drought simulation in yeast

First, prepare SD/-Ura solid medium containing 1 and 2 M mannitol (add 18.217 g mannitol to 100 ml SD/-Ura solid medium, and the mannitol concentration is 1 M). Pick up the streaked bacteria on the plate, and add them to SD/-Ura liquid medium for propagation on a 28 ℃ shaker. Adjust the initial concentration of the bacterial solution to OD_600_ = 1.0, and then gradient dilute it according to 10^−1^, 10^−2^, 10^−3^, and 10^−4^. Take 2 μL of the diluted bacterial solution, and drop it on the SD/-Ura solid medium containing mannitol and without mannitol; make 4 replicates for each concentration, and place it at 28 ℃ for 2 to 3 d to observe the growth of yeast.

### RNA extraction, reverse transcription, and real-time PCR

For glasshouse experiments, total RNA was extracted from the second true leaf of the cotton material with 3 biological replicates using Trizol reagent (Invitrogen, United States). After DNase I (Promega, United States) treatment, total RNA (5 μg for each sample) was reverse transcribed using a FastQuant RT Kit (Tiangen Biotech Co. Ltd., China) according to the manufacturer's protocol. Real-time PCR analysis was carried out using the Bio-Rad CFX ConnectTM Real-Time PCR Detection System (Hercules, United States) with SYBR Green SuperReal PreMix Plus (Tiangen Biotech Co. Ltd., China). PCR conditions were 95 °C for 15 min, followed by 40 cycles of 95 °C for 10 s and 60 °C for 30 s. Three biological replicates were used with 3 technical replicates. Relative gene expression levels were determined using the 2^−ΔΔCT^ method. The gene-speciﬁc primers used for RT-qPCR were designed using the Primer Database (Lu et al. 2018), which were listed in Table S1. The Cotton Histone 3 (AF024716) gene was used as a reference gene to normalize the cDNA samples.

### Protein extraction and protein gel blot analysis

Protein was extracted from 21-d-old cotton seedlings with an extraction buffer containing 50 mM Tris-HCl, pH 7.5, 150 mM NaCl, 0.1% NP40, 4 M urea, and 1 mM PMSF. To detect GhGGP1 protein, the custom polyclonal anti-GhGGP1 antibody used in this study was generated by Younuoke Biotechnology (Suzhou, China). Briefly, a recombinant polypeptide covering amino acids 1–178 of GhGGP1 was constructed into the pET28a vector, expressed and purified, then conjugated and immunized into New Zealand rabbits to produce crude antiserum. The antiserum was further purified by peptide affinity chromatography to remove nonspecific immunoglobulins. For immunoblot analysis, the purified primary anti-GhGGP1 antibody was applied at a dilution of 1:3000. The commercial anti-FLAG antibody (Engibody, Cat. No. AT0059) was used at a 1:10,000 dilution. Horseradish peroxidase-conjugated goat anti-rabbit IgG (PTMab, Cat. No. PTM-6261) served as the secondary antibody, and chemiluminescent signals were captured using enhanced chemiluminescence substrate (Millipore).

### AsA measurement

For routine determination of ascorbic acid (AsA) content in a large number of samples, an ascorbic acid assay kit (Solarbio, Beijing, China) was used. To precisely verify the significant increase in AsA content detected in the *uorf_GhGGP1_* mutants, AsA levels in wild-type (WT) and 4 homozygous mutant lines were quantified by Sanshu Biotechnology using Ultra-High-Performance Liquid Chromatography coupled with a Q Exactive Orbitrap high-resolution tandem mass spectrometer (UHPLC-QE HRMS; Thermo Fisher Scientific).

### Drought stress tolerance assays

During the vegetative stage, 5-wk-old genetically modified cotton seeds and wild-type cotton were cultivated in pots and adequately watered. Then, water was withheld completely (drought treatment). The plants were subsequently rewatered regularly to recover.

### Malondialdehyde (MDA) content and total antioxidant capacity (T-AOC) activity measurements

MDA content in the second true leaf of cotton was measured with the 2-thiobarbituric acid (TBA) method using a MDA assay kit (Jiancheng Bioengineering Institute, Nanjing, China) according to the manufacturer's protocol. The second true leaf of cotton T-AOC activity measurement was performed with the ferric reducing antioxidant power (FRAP) method using a T-AOC assay kit (Jiancheng Bioengineering Institute) according to the manufacturer's protocol. Three replicates were taken for each treatment group before following the assay protocol to obtain values.

### The 3,3'-diaminobenzidine (DAB) and nitroblue tetrazolium (NBT) staining

The leaves of cotton under mock and 15% PEG 6000 treatment conditions were detached for staining. For DAB staining, the samples were immersed in the DAB solution (Solarbio Life Science, Beijing, China) for 1 night and subsequently treated in 95% (v/v) ethanol for decolorization. For NBT staining, the samples were immersed in NBT staining solution (Solarbio Life Science, Beijing, China) for 1 night and subsequently treated with 95% (v/v) ethanol for decolorization.

### Identification of drought resistance in the field

In the cement pond with a length of 15 m, a width of 2 m, and a depth of 25 cm, each material has 1 row of the area and 3 replicates, and the 3% seedling-stage repeated drought method is used for unified identification and grading (high resistance, resistance, tolerance, and intolerance).

### Yeast one-hybrid assay

Yeast one-hybrid assays of LacZ were performed to characterize the interaction between *GhBBM* and the promoter of *GhGGP1*. The CDS of *GhBBM* was inserted into the pB42AD vector, which was linearized by EcoRI/XhoI digestion. The putative *GhBBM*-binding motif in the *GhGGP1* promoter sequence was selected and cloned into the KpnI-SalI-digested pLacZi-2u. The pB42AD, pB42AD: *GhBBM*, and pro*GhGGP1*-A01:LacZ, mpro*GhGGP1*-A01:LacZ were separately transformed into the EGY48 strain, and the transformants were selected on the SD/Trp/-Ura medium for 2 to 3 days at 28 °C. Next, the transformants were selected on the SD/Trp/-Ura/X-gal medium with rafﬁnose and galactose. The activity of galactosidase was determined using the ONPG method. The primers used are listed in Table S1.

### Electrophoretic mobility shift assay

For electrophoretic mobility shift assay (EMSA), the assay was performed using an EMSA kit (Thermo Fisher Scientiﬁc, Shanghai, China) following a previously described method (Hellman and Fried 2007). An EMSA was performed to determine whether *GhBBM* could regulate *GhGGP1* expression. The CDS of *GhBBM* was inserted into the pCZN1 vector with a His label, which was linearized by NdeI/EcoRI digestion. The resulting pCZN1-His-*GhBBM* expression vector was transformed into the *E. coli* strain Rosetta (DE3.1). The pCZN1-His empty vector was used as a negative control. Protein expression was induced by 0.2 mM IPTG in LB medium with shaking at 15 °C overnight. The EMSA was performed using the LightShift Chemiluminescent EMSA Kit following the manufacturer's instructions (Thermo Fisher Scientiﬁc). Biotin-labeled oligonucleotide probes were synthesized by BGI (Beijing, China). The primers and probes used in EMSA are listed in Table S1. The images were scanned with the ChemiScope 6000 chemiluminescence imaging system (Clinx, Shanghai, China) according to the manufacturer’s instructions.

### Chromatin immunoprecipitation combined qPCR assay (ChIP-qPCR)

Two-week-old cotton transgenic seedlings were harvested for analysis using the ChIP-qPCR assay as previously described (Li et al. 2018a). A polyclonal anti-GFP antibody was used for the ChIP assay, with an IgG antibody as a negative control. Briefly, the chromatin was extracted and sonicated to obtain an average DNA fragment size of 0.2 to 1.0 kb. The DNA fragments specifically associated with the TaERFL1a gene were released, purified, and used as templates for qPCR with a specific primer (Table S1).

### Acid fuchsin staining tracing test

Dye-tracing assays were performed as follows. When plants of each genotype had reached the 2-leaf stage, 3-cm stem segments were excised from above the cotyledonary node. The segments were immersed in 1 of 2 solutions: (i) a control solution containing 0.1% (w/v) acid fuchsin in water or (ii) a drought-treatment solution containing 0.1% (w/v) acid fuchsin and 10% (w/v) PEG6000. After incubation for 16 h, we longitudinally sectioned the stem segments and photographed the acid fuchsin dye transport distance using a Canon camera.

### Relative water content (RWC) measurement

RWC was determined as follows. Cotton seedlings at the 2-leaf stage were treated with 10% (w/v) PEG6000 in hydroponic solution for 12 h. After removal from the solution, the seedlings were blotted dry, and their fresh weight (FW) was immediately recorded. The seedlings were then incubated in darkness for 24 h to allow full hydration, blotted dry, and weighed to obtain turgid weight (TW). The samples were oven-dried at 105 °C for 15 min to inactivate enzymes, followed by drying at 65 ℃ to constant weight (ie until the difference between 2 consecutive measurements was ≤0.002 g). After cooling, the dry weight (DW) was recorded. RWC was calculated as RWC (%) = [(FW − DW)/(TW − DW)] × 100%.

### Statistical analyses

All experiments were conducted with at least 3 biological replicates. Student's *t*-test and one-way ANOVA were used to determine statistical signiﬁcance (*P < 0.05; **P < 0.01; ***P < 0.001).

## Results

### Mutating the *GhGGP1* uORF enhances the translation of downstream pORF

Literature studies have shown that in Arabidopsis, VTC2 (AT4G26850) and its homologous gene VTC5 (AT5G55120) jointly regulate AsA synthesis, with VTC2 being the main player (*vtc2* mutants have 10% to 30% wildtype AsA) and VTC5 (*vtc5* mutants have ∼80% wildtype AsA) playing a lessor, more tissue specific role (Conklin et al. 2000). The Arabidopsis double mutant (*vtc2/vtc5*) causes almost complete loss of AsA resulting in growth arrest immediately upon germination, but the seedlings can be rescued by exogenous AsA or L-galactose supplementation (Dowdle et al. 2007). We conducted a phylogenetic analysis of *GhGGP1* and several other homologous genes, which showed that *GhGGP1* is the closest homolog of *AtGGP1* and *ZmGGP1* (Fig. 1a). This alignment shows highly conserved regions within the proteins, as well as the conserved histidine triad (HIT) motif of the HIT protein superfamily, the conserved KKRP nuclear localization signal (Fig. 1b, Figure S1). On the cottonfgd website (https://cottonfgd.org/), 7 GGP1-like genes were identified. Among the 7, *GhGGP1-D05a* showed the lowest similarity to the protein sequence of *AtVTC2*. The sequence comparisons between the 7 *GhGGP1* proteins and the *AtVTC2* protein revealed that they share between 68.81% and 70.58% identity (Figure S2a). The *GhGGP1* homoeologs are broadly expressed across multiple tissues, suggesting possible roles of this gene in plant development (Figure S2b). For *GhGGP1-A01*, *GhGGP1-D01*, *GhGGP1-A04*, *GhGGP1-D05b*, *GhGGP1-A13*, and *GhGGP1-D13* genes, a highly conserved uORF (similarity reaches 93.69%) was identified in the long 5'UTR of the mRNA initiating from the non-canonical ATC (isoleucine) start codon. This uORF encodes a small peptide of 58 to 59 amino acids, which inhibits the translation of GhGGP1 when cellular AsA levels are too high, thereby downregulating AsA synthesis through a negative feedback mechanism (Fig. 1c and d).

**Figure 1:**
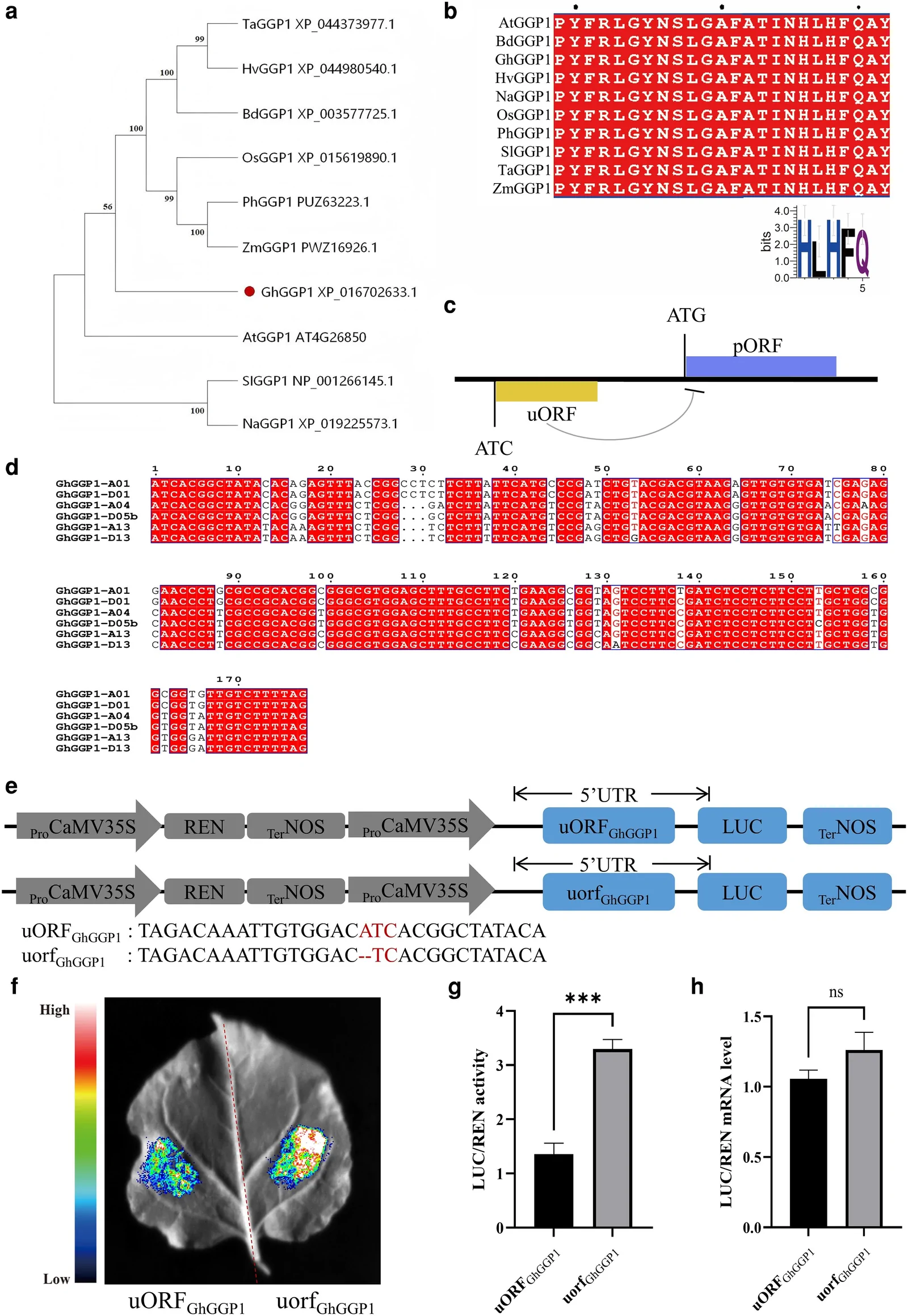
Sequence analysis of *GhGGP1* and functional validation of uORF_GhGGP1_. a) Unrooted phylogenetic tree of GGP1 in different species, Ta: *Triticum aestivum*, Hv: *Hordeum vulgare*, Bd: *Brachypodium distachyon*, Os: *Oryza sativa*, Ph: *Panicum hallii*, Zm: *Zea mays*, Gh: *Gossypium hirsutum*, At: *Arabidopsis thaliana*, Sl: *Solanum lycopersicum*, Na: *Nicotiana attenuata*. The value of bootstrap is 1000. A marked dot indicates *GhGGP1*. b) Amino acid sequence alignment and conserved domain analysis of GGP1 proteins from a range of species. c) Gene structure of *GhGGP1.* The uORF is located at the 5'UTR region. d) Deoxyribonucleic acid sequence alignment of *GhGGP1* uORFs. Background shading represents 100% conservation of deoxyribonucleic acid between uORFs. e) Schematics of normal uORF_GhGGP1_ or mutant uORF_GhGGP1_ (uorf_GhGGP1_) involved the mutation of the uORF initiation codon to -TC. f) *N. benthamiana* transient expression assay of normal uORF_GhGGP1_ and mutant uORF_GhGGP1_ luciferase activity. g, h) LUC/REN activity (f) and mRNA level (g) in dual-luciferase assays. The mean LUC/REN activity and mRNA levels conferred by normal uORF_GhGGP1_ or mutant uORF_GhGGP1_ were normalized to those of the control. Data represent the mean ± SD of 15 sample measurements from triplicate experiments. ***P < 0.001; ns, no significant difference by two-tailed Student's *t*-test.

Next, we validated whether mutating the region harboring a *GhGGP1-A01* uORF initiation codon increases the translation of a downstream pORF mRNA. The read-out for translation was a dual-luciferase reporter system (Fig. 1e). The normal 5'UTR, with intact uORF, and 1 mutant 5'UTR (-TC, uORF initiation codon mutated from ATC to -TC to prevent uORF translation) were each cloned upstream of the LUC coding region in an expression cassette driven by the 35S promoter (Figure S3). The resulting constructs also harbored a second 35S-promoter-driven cassette expressing REN as an internal vector control. Analysis of LUC activity indicated that uORF_GhGGP1_ directly activated the LUC activity (Fig. 1f). Two constructs for *GhGGP1* were expressed in *N. benthamiana* leaves, and we calculated the mean LUC/REN activities and LUC/REN mRNA levels using data from multiple assays (Fig. 1g and h). The 5'UTR with the uORF initiation codon mutated to -TC was effective for increasing LUC/REN activity. Quantitative RT-PCR assays revealed that the LUC/REN mRNA levels transcribed from the various constructs did not differ significantly. These data indicate that small deletions that remove the uORF initiation codon might be generally effective at upregulating the translation of pORF transcripts without affecting the transcription of the pORF. Thus, we chose regions harboring the uORF initiation codons as target sites for single-guide RNAs (sgRNAs) in subsequent genome editing experiments.

### *GhGGP1* protein was upregulated in the *uorf_GhGGP1_* mutants

We edited uORF_GhGGP1_ via the CRISPR/Cas9 system, and the targeted sites were located on the initiation codon ATC of uORF (Fig. 2a, Figure S4). Then, 4 homozygous transgene-free lines (*uorf_GhGGP1_-1*, *uorf_GhGGP1_-2*, *uorf_GhGGP1_-3*, and *uorf_GhGGP1_-4*) of cotton were produced, and comprehensive analyses confirmed the absence of off-target effects associated with this editing event (Fig. 2b, Figure S5 and Figure S6). Transcript levels did not differ between WT and mutant plants (Fig. 2c), but *GhGGP1* protein was massively upregulated in mutants, particularly in *uorf_GhGGP1_-1* (Fig. 2d). Meanwhile, compared with WT plants, the *uorf_GhGGP1_* mutant plants exhibited varying degrees of AsA content, with all being very large at 17.1-, 9.5-, 4.3-, and 6.2-fold, respectively (Violet ultraviolet spectrophotometry) (Figure S13). Given that spectrophotometry may suffer from nonspecific substrate interference and compromise detection accuracy, we precisely quantified AsA using UHPLC-QE HRMS. The results consistently confirmed a substantial accumulation of AsA in all mutant lines (Fig. 2e), supporting the reliability of the original measurements obtained by spectrophotometric assay. The UHPLC-QE HRMS quantification showed that the AsA content of the *uorf_GhGGP1_* mutant is 4.03 to 14.79 times higher than that of the WT. Subsequently, we employed 4 independent T3 homozygous cotton seedling lines (*uorf_GhGGP1_-1*, *uorf_GhGGP1_-2*, *uorf_GhGGP1_-3*, and *uorf_GhGGP1_-4*) to analyze the mechanism by which *GhGGP1* regulates drought tolerance in cotton.

**Figure 2:**
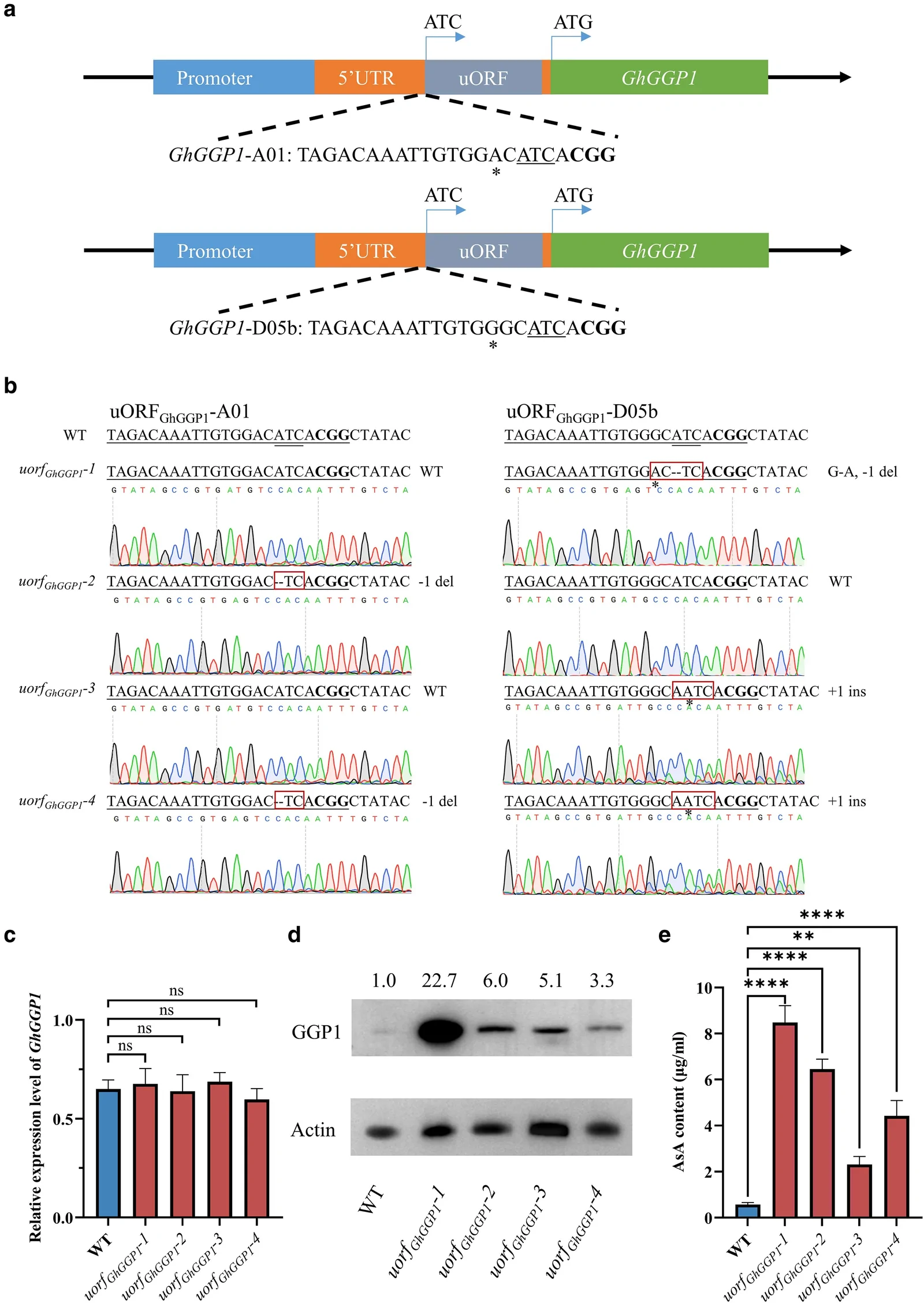
Effect of editing the uORF of *GhGGP1*. a) Schematic maps of the sgRNA targeted sites on the uORF region of *GhGGP1*. Underlined bases, initiation codon of the uORF_GhGGP1_; bold bases, PAM motifs (NGG); *, base differences between the 2 target sequences *GhGGP1*-A01 and *GhGGP1*-D05b. b) Sequencing results and chromatograms of the *GhGGP1* homozygous mutant lines from the T1 generation. Bold bases, PAM motif; double-underlined bases, initiation codon of the uORF_GhGGP1_; underlining, target sequence; boxes, regions where base changes occur; dashes, deletions; and bases marked with asterisks, insertions and substitutions. c) Comparison of mRNA levels among WT control, *uorf_GhGGP1_-1*, *uorf_GhGGP1_-2*, *uorf_GhGGP1_-3*, and *uorf_GhGGP1_-4*. Histone was used as an internal control for the quantitative real-time PCR assay. Data represent mean ± SD for 3 independent experiments (*n* > 10). ns, with no significant difference by one-way ANOVA. d) Comparison of *GhGGP1* protein levels among WT control, *uorf_GhGGP1_-1*, *uorf_GhGGP1_-2*, *uorf_GhGGP1_-3*, and *uorf_GhGGP1_-4*. Actin was used as a loading control in the immunoblot assay. e) AsA contents were independently quantified by ***UHPLC-QE HRMS***. Data represent mean ± SD for 3 independent experiments (*n* > 10). **P < 0.01, ****P < 0.0001 by one-way ANOVA.

### *GhGGP1* positively regulates the drought resistance of cotton

We first compared the growth status of recombinant yeast INVSc1 (pYES2*-GhGGP1*) and control yeast INVSc1 (pYES2) when mannitol is added and when it is not added. The results showed that with the gradual increase of mannitol concentration, the growth status of yeast carrying pYES2*-GhGGP1* recombinant plasmid was significantly better than that of control yeast, which preliminarily confirmed that *GhGGP1* played a role in the response to drought stress (Figure S7).

For drought stress, the WT and the 4 *uorf_GhGGP1_* mutant seeds were treated with 10% PEG6000. Compared with WT seeds, the 4 *uorf_GhGGP1_* mutant seeds presented higher germination rates and longer root lengths, whereas there were no significant differences in these parameters under normal conditions (Fig. 3a to d). Five-week-old WT and the 4 *uorf_GhGGP1_* mutant cotton plants were subjected to 15 d of drought treatment. As shown in Fig. 3e, compared with the 4 *uorf_GhGGP1_* mutant plants, the WT cotton plants presented more severe leaf curling and wilting. Rewatering for 3 d, the survival rates of the 4 *uorf_GhGGP1_* cotton plants were significantly higher than that of the WT plants (Fig. 3e). Furthermore, DAB and NBT histochemical staining analysis revealed that, compared with WT plants, the 4 *uorf_GhGGP1_* cotton plants presented lighter staining after 15 d of drought treatment (Fig. 3f). To explore the physiological mechanisms of *uorf_GhGGP1_* mutant lines in response to drought stress tolerance, the levels of MDA, ASA, and T-AOC were quantified (Fig. 3g to i). Under well-watered conditions (0 h), the MDA content did not significantly differ between the *uorf_GhGGP1_* mutant lines and the WT. However, after 1 h of PEG6000 treatment, the leaves showed a stress response, and the MDA content in both types of materials decreased to the lowest value and then showed an upward trend, but the MDA content of the *uorf_GhGGP1_* gene editing material increased slowly, and after 24 h, its MDA content was lower than that of the WT (Fig. 3g). Moreover, the contents of AsA and the affinity of T-AOC were significantly higher in *uorf_GhGGP1_* mutant lines than in the WT under well-watered conditions. After 1 h of PEG6000 treatment, the AsA content in both types of materials reached the highest value, and the AsA content of the *uorf_GhGGP1_* gene editing material was significantly stronger than that of the WT. The trend of T-AOC changes is consistent with AsA (Fig. 3h and i).

**Figure 3:**
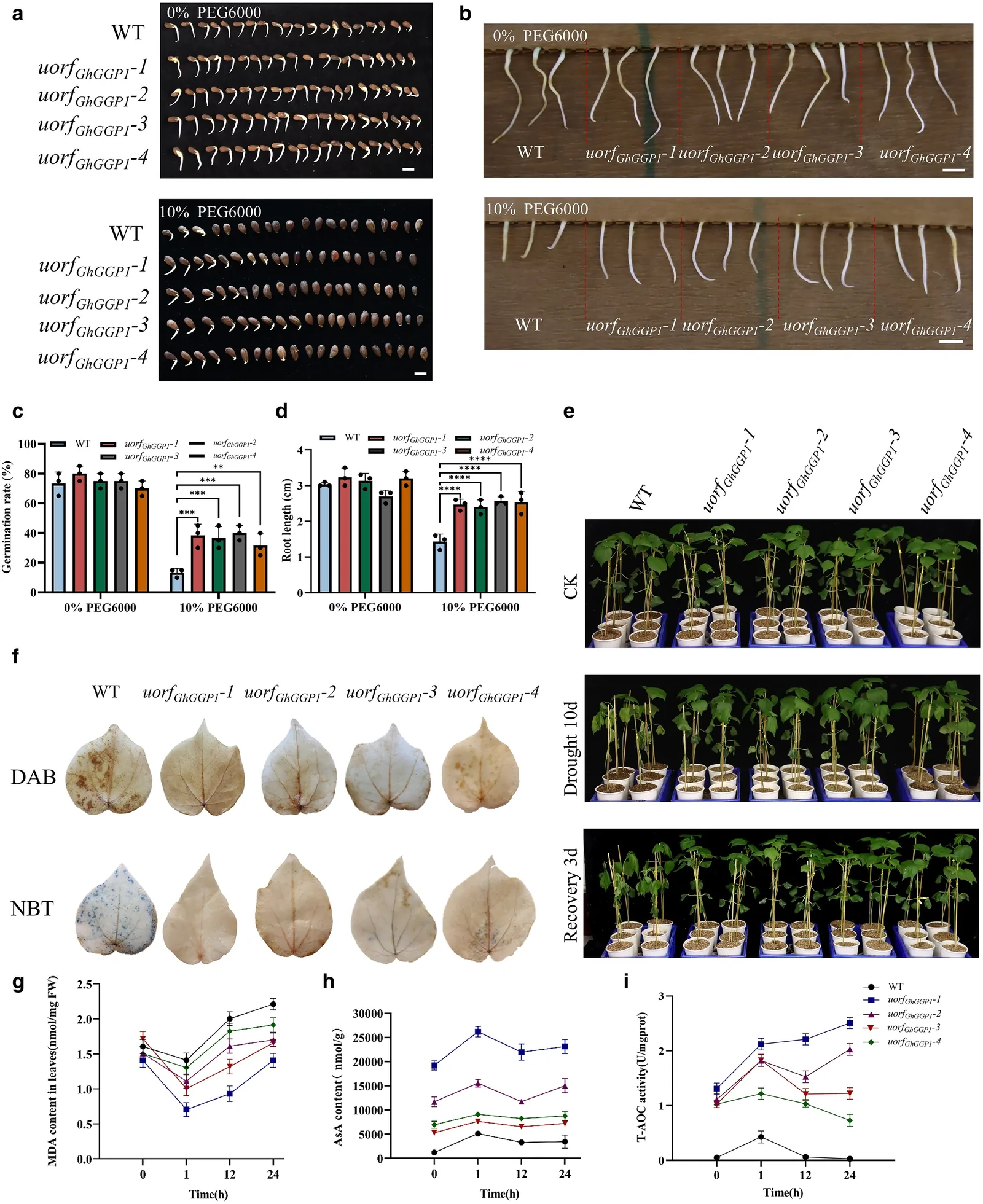
Overexpression of *GhGGP1* promoted the drought tolerance of cotton. a) Representative phenotypes of seed germination in the WT and *uorf_GhGGP1_* mutant cotton seeds with or without PEG6000 treatment. Bars, 1 cm. b) Representative phenotypes of root length in the WT and *uorf_GhGGP1_* mutant cottons with or without PEG6000 treatment. Bars, 1 cm. c) Germination rate in WT and *uorf_GhGGP1_* mutant cotton seeds under PEG6000 treatment. The error bars in a and c indicate the mean ± SD of 3 independent biological replicates (*n* = 40). **P < 0.01, ***P < 0.001 by one-way ANOVA. d) Root length in WT and *uorf_GhGGP1_* mutant cottons under PEG6000 treatment. The error bars in b and d indicate the mean ± SD of 3 independent biological replicates (*n* = 15). *P < 0.05 by one-way ANOVA. e) Representative phenotypes of 5-wk-old WT and *uorf_GhGGP1_* mutant cotton plants after drought treatment for 15 d. The plants were subsequently rewatered for 3 d to allow them to recover. Bars, 2 cm. f) Representative photographs showing DAB (up) and NBT (down) histochemical staining of leaves from 5-wk-old WT and *uorf_GhGGP1_* mutant cotton plants after drought treatment for 15 d. g to i) MDA content, reduced AsA content, and T-AOC in WT and *uorf_GhGGP1_* mutant cotton plants after drought treatment for 15 d.

We also evaluated the drought resistance of the *uorf_GhGGP1_* mutant plants in the field. The relative survival rate of the *uorf_GhGGP1_* mutant plants was 42.91% to 54.20%, and the relative survival rate of the wild-type material ZM24 was 34.71%. The *uorf_GhGGP1_* mutant plants had good drought resistance (Figure S8). These results suggest that the overexpression of *GhGGP1* induces physiological changes in cotton, enhancing drought tolerance in cotton.

### *GhBBM* acts as a transcriptional activator of *GhGGP1*

To identify and characterize upstream regulatory factors of *GhGGP1*, we input the full upstream regulatory sequence of *GhGGP1* (including the promoter and 5'UTR harboring the uORF) into the PlantRegMap website (https://plantregmap.gao-lab.org/), and the analysis shows that *GhBBM* is the most possible transcription factor upstream of *GhGGP1*. *GhBBM* belongs to the APETALA2/ETHYLENE RESPONSIVE FACTOR (AP2/ERF) transcription factor, which plays crucial roles in plant growth, development, and responses to biotic and abiotic stresses (Boutilier et al. 2002; Kong et al. 2023). It was demonstrated that osmotic stress-induced *PtoERF15* and its target gene *PtoMYC2b* are involved in mediating vessel size, density, and cell wall thickness in response to drought in poplar. Overexpression of *PtoERF15* contributes to the maintenance of stem water potential, thereby increasing drought tolerance (Kong et al. 2023). In order to verify if *GhGGP1* is regulated by *GhBBM*, we ﬁrst conducted a dual-LUC assay. Separate vectors containing the Cauliflower mosaic virus-derived 35S promoter driving *GhBBM* and the *GhGGP1* promoter driving LUC were co-transfected in tobacco and displayed a much higher relative LUC/REN ratio than the negative control in a dual-LUC assay, supporting interaction (Fig. 4a to c). The cis-elements of *GhBBM* were predicted by JASPAR. Among many predicted *GhBBM* cis-elements, an ethylene-response element was identiﬁed within the uORF of *GhGGP1-A01* (−341 bp, CTTATTCATGCC). Y1H assay showed that *GhBBM* could speciﬁcally bind to the *GhGGP1-A01* promoter, and such speciﬁc binding was abolished when the motif was mutated (Fig. 4d to f). To verify the binding speciﬁcity of *GhBBM* to the motif, we carried out EMSAs and found that *GhBBM*-HIS fusion proteins could bind DNA probes containing the motif, whereas non-labeled competing probes effectively reduced the binding ability of *GhBBM* in a dose-dependent manner, and mutation of the core sequence abolished the binding (Fig. 4g). Next, we performed ChIP-qPCR analysis using an anti-FLAG antibody. We found that the motif was significantly enriched (Fig. 4h). These results indicate that *GhBBM* binds directly to the *GhGGP1* promoter and acts as a transcriptional activator of *GhGGP1*.

**Figure 4:**
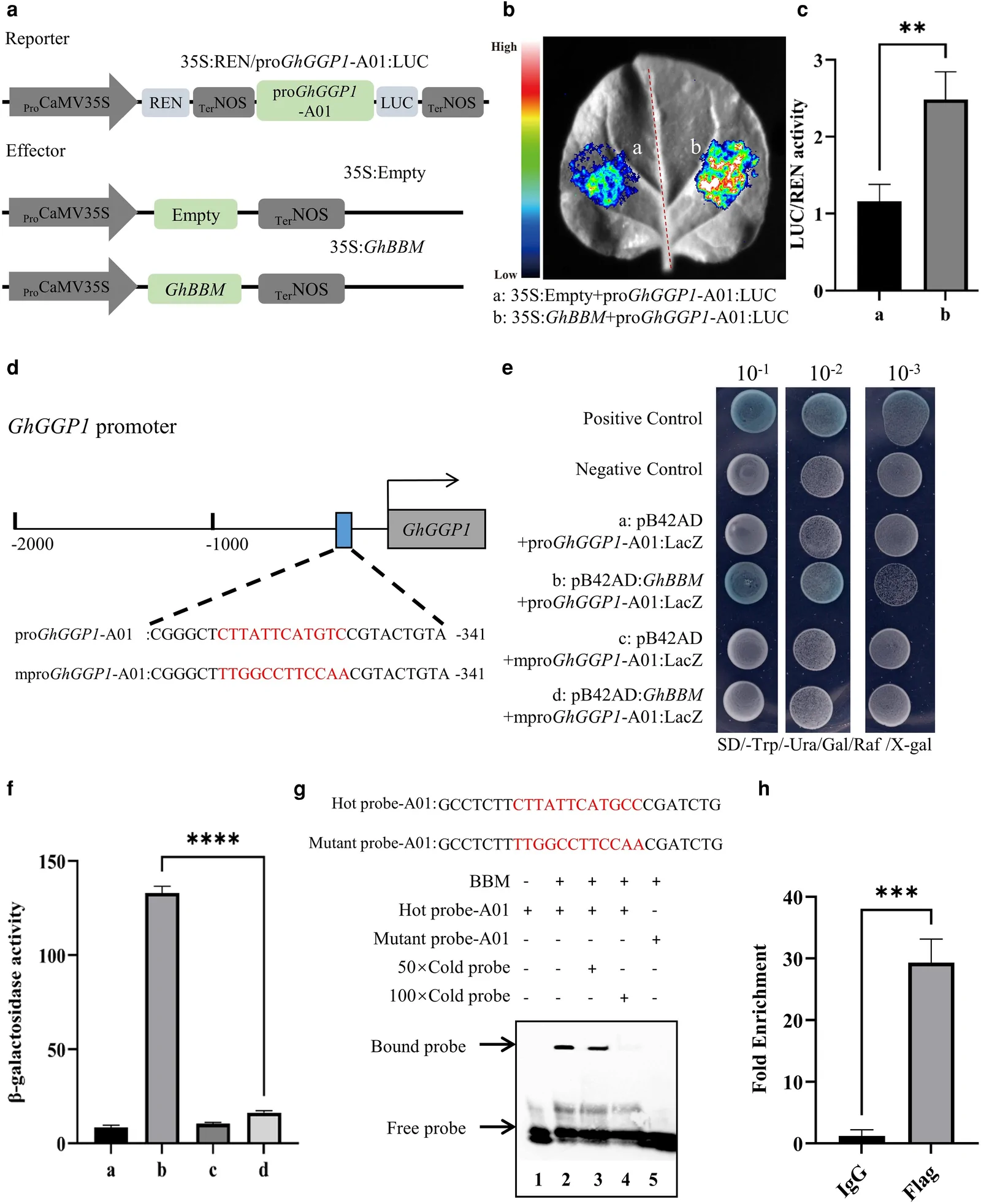
*GhBBM* binds to the promoter of *GhGGP1* and activates its transcription. a to c) Luciferase reporter assay. *GhBBM* can activate transcription of the *GhGGP1*-A01 promoter in *N. benthamiana* leaves. Bioluminescence signals were imaged and quantified with the Tanon 5200 Multi Chemiluminescent Imaging System to indicate the luciferase activities. The error bars in c indicate the mean ± SD of 3 independent biological replicates (*n* = 10). **P < 0.01 by Student's *t*-test. d to f) Yeast one-hybrid assay. The *GhGGP1*-A01 promoter is responsible for the interaction with *GhBBM*. Yeast cells were grown on SD/-Trp/-Ura medium with X-gal. g) EMSA assay. The biotin-labeled WT (hot) and mutated (mutant) probes are indicated. Competition analysis was conducted with excess unlabeled WT probes (cold) at 50× and 100× amounts of hot probes. h) ChIP-qPCR signals were normalized to a negative control. ***P < 0.001 by Student's *t*-test.

### *GhBBM* is a positive factor that regulates the gene of *GhGGP1* and AsA synthesis

In order to explore the *GhBBM* expression patterns in different tissues of cotton, we obtained transcriptome data of 2 copies of *GhBBM* from the CottonMD website (https://yanglab.hzau.edu.cn/CottonMD/). The expression data indicated that *GhBBM* is only expressed in the stem, so we used the stem as the sampling location (Figure S9). In order to gain a further understanding of the regulatory roles of *GhBBM*, 2 stable overexpression transgenic cotton lines were generated (Figure S10). qRT-PCR conﬁrmed that the 2 independent transgenic lines accumulated high levels of *GhBBM* transcripts (4.06- and 5.43-fold, respectively; Figure S11a), which in turn increased the expression levels of *GhGGP1* by 2- and 4.5-fold (Figure S11b). AsA content increased by 2.51- and 3.48-fold in comparison to those of controls, respectively (Figure S11c). Using the CRISPR/Cas9 system, we further mutated *GhBBM* in *Gossypium hirsutum* L. The 2 targeted sites were located within the ﬁrst and second exons of 2 copies of *GhBBM*; sgRNA2 was not edited after subsequent testing (Figure S11d). In total, 2 independent *GhBBM* mutants (KO-*GhBBM*-1, a 1-bp deletion in *GhBBM*-A08 and a 1-bp deletion in *GhBBM*-D08; KO-*GhBBM*-2, a 1-bp insertion in *GhBBM*-A08 and a 1-bp deletion in *GhBBM*-D08) (Figure S11e) were identiﬁed. qRT-PCR demonstrated that the expression of *GhGGP1* was decreased in *GhBBM*-edited lines (Figure S11f). Meanwhile, the AsA contents of the *GhBBM*-edited lines were decreased compared with the control group (Figure S11g). Taken together, these ﬁndings support that *GhBBM* is an upstream regulatory gene of *GhGGP1* and a positive factor that modulates AsA synthesis.

### *GhBBM* positively regulates drought tolerance in cotton

To evaluate the contribution of *GhBBM* to drought tolerance in cotton, the WT, OE-*GhBBM*, and KO-*GhBBM* seeds were treated with 10% PEG6000. Compared with WT seeds, the OE-*GhBBM* seeds presented higher germination rates and longer root lengths, while the KO-*GhBBM* seeds presented lower germination rates and shorter root lengths, and there were no significant differences in these parameters under normal conditions (Fig. 5a to f). To investigate whether stem-specifically expressed *GhBBM* affects whole-plant drought responses by modulating water transport, we performed dye-tracing assays under drought stress. The results showed that, compared with the wild type, OE-*GhBBM* lines exhibited enhanced water transport capacity, whereas KO-*GhBBM* lines showed reduced capacity. Similarly, under the regulation of *GhBBM*, the ggp1 mutant also displayed greater transport capacity than the wild type (Figure S12a and b). These results indicate that *GhBBM* is involved in modulating stem water transport under drought conditions. In addition, under drought stress, OE-*GhBBM* plants exhibited significantly higher relative water content than wild-type and KO-*GhBBM* plants, and the *uorf_GhGGP1_* mutant also showed significantly higher relative water content than the wild type (Figure S12c and d). These results indicate that enhanced stem water transport capacity contributes to maintaining leaf water status.

**Figure 5:**
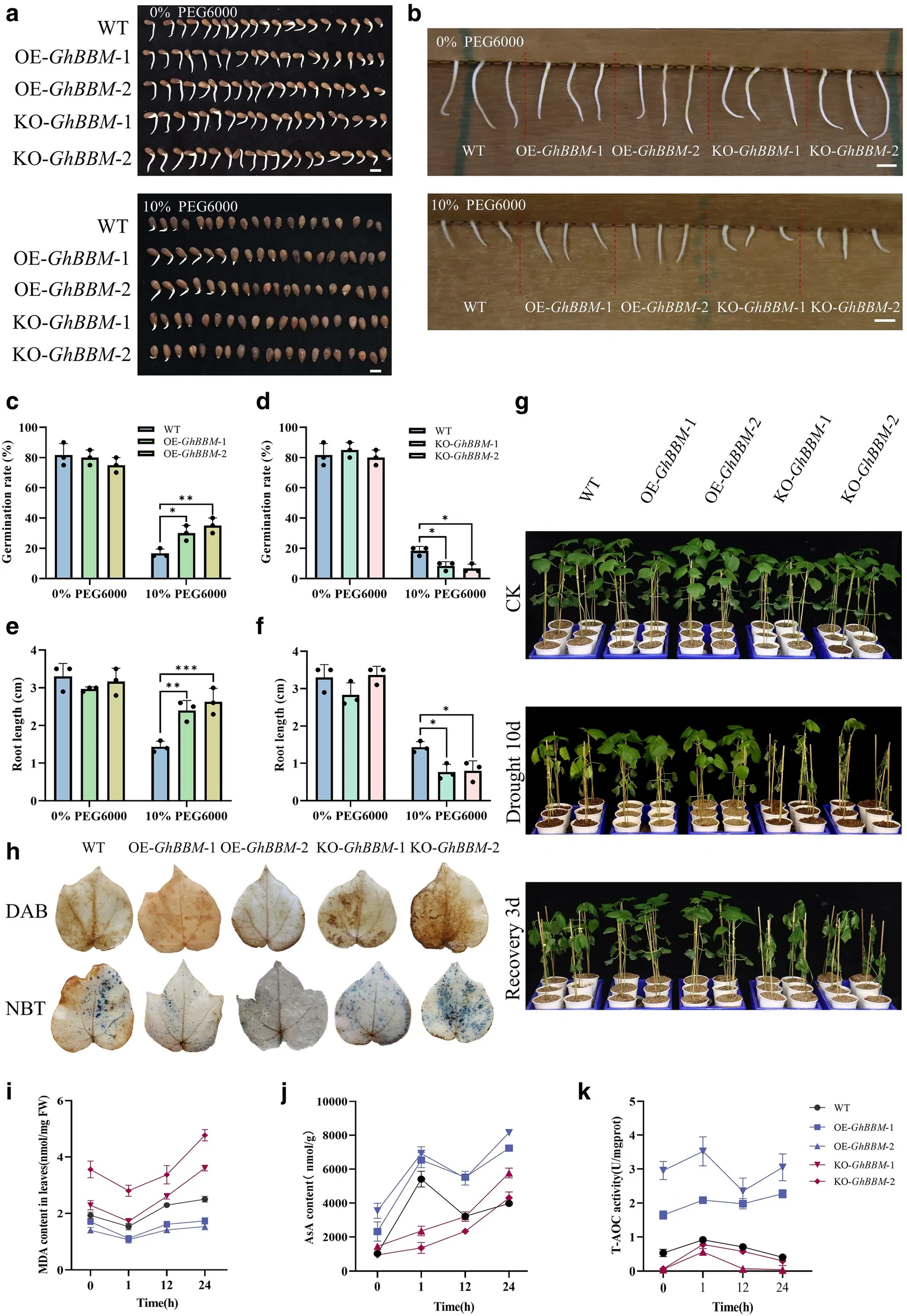
Identification of drought resistance for the WT control, the OE-*GhBBM* lines, and the KO-*GhBBM* lines. a) Representative phenotypes of seed germination in the WT, OE-*GhBBM*, and KO-*GhBBM* transgenic cotton seeds with or without PEG6000 treatment. Bars, 1 cm. b) Representative phenotypes of root length in the WT, OE-*GhBBM*, and KO-*GhBBM* transgenic cottons with or without PEG6000 treatment. Bars, 1 cm. c, d) Germination rate in WT, OE-*GhBBM*, and KO-*GhBBM* transgenic cotton seeds under PEG6000 treatment. The error bars in a, c, and d indicate the mean ± SEs of 3 independent biological replicates (*n* = 20). e, f) Root length in WT, OE-*GhBBM*, and KO-*GhBBM* transgenic cottons under PEG6000 treatment. The error bars in b, e, and f indicate the mean ± SEs of 3 independent biological replicates (*n* = 15). g) Representative phenotypes of 5-wk-old WT, OE-*GhBBM*, and KO-*GhBBM* transgenic cotton plants after drought treatment for 15 d. The plants were subsequently rewatered for 3 d to allow them to recover. Bars, 2 cm. h) Representative photographs showing DAB (up) and NBT (down) histochemical staining of leaves from 5-wk-old WT, OE-*GhBBM*, and KO-*GhBBM* transgenic cotton plants after drought treatment for 15 d. i to k) MDA content, reduced AsA content, and T-AOC in WT, OE-*GhBBM*, and KO-*GhBBM* transgenic cotton plants after drought treatment for 15 d. Note that each experiment of c to f was performed in 3 replicates. All of the above error bars denote standard deviation (±SD). Significant differences to WT were detected by one-way ANOVA using GraphPad Prism 8: *P < 0.05; **P < 0.01; ***P < 0.001.

Five-week-old WT, OE-*GhBBM*, and KO-*GhBBM* cotton plants were subjected to 15 d of drought treatment. As shown in Fig. 5g, compared with the WT cotton plants, the OE-*GhBBM* cotton plants presented less severe leaf curling and wilting, while the KO-*GhBBM* cotton plants showed a drought-sensitive phenotype with necrotic leaves and a burning sensation. Rewatering for 3 d, the survival rates of the OE-*GhBBM* cotton plants were significantly higher than that of the WT plants, and the survival rates of the KO-*GhBBM* cotton plants were significantly lower than that of the WT plants (Fig. 5g). Furthermore, DAB and NBT histochemical staining analysis revealed that, compared with WT plants, the OE-*GhBBM* cotton plants presented lighter staining and the KO-*GhBBM* cotton plants presented deeper staining after 15 d of drought treatment (Fig. 5h). The measurement results of physical and chemical indicators MDA, AsA, and T-AOC are consistent with the observed phenotype (Fig. 5i–k). In summary, *GhBBM* exerts a positive influence on the drought tolerance of cotton.

## Discussion

In cotton, drought stress severely limits yield and fiber quality; however, improving drought resistance remains challenging due to complex genetic architectures and trade-offs between growth and stress responses. *GhGGP1*, a key enzyme in ascorbate biosynthesis, plays a central role in redox homeostasis and drought adaptation. Direct manipulation of its coding region, however, may disrupt metabolic balance and developmental stability. Within this context, targeting the uORF of *GhGGP1* emerges as an alternative strategy to enhance stress tolerance by modulating protein abundance while preserving endogenous transcriptional and metabolic networks. Therefore, uORF editing offers a practical and environmentally considerate precision-breeding strategy aimed at improving drought resistance in cotton.

uORFs have been demonstrated to function as effective regulators that suppress protein translation at the initiation stage (Mou et al. 2025; Nazarian-Firouzabadi et al. 2025; Zhang et al. 2025a, 2025b). Previous studies have demonstrated that targeting the uORF of VTC2 by gene editing is a feasible strategy to increase ascorbic acid concentration in plants. For example, CRISPR/Cas9 targets the uORF sequence of *SlVTC2*, and the mutation disrupts its function, increasing the ascorbic acid concentration in leaves by about 1.4-fold (Li et al. 2018b). Similarly, CRISPR/Cas9 targeted the uORF of *AtGGP11* in *Arabidopsis thaliana* and the uORF of *LsGGP11* and *LsGGP12* in lettuce, increasing the concentration of ascorbic acid in leaves by 1.7-fold, 1.4-fold, and 2.6-fold, respectively. In addition, the increased concentration of ascorbic acid in lettuce enhanced tolerance to oxidative stress induced by the oxidant paraquat (Zhang et al. 2018).

In this study, we used CRISPR/Cas9 to accurately target the uORF sequence of *GhGGP1*, which avoided producing additional proteins, and obtained the *uorf_GhGGP1_* gene-edited material. The AsA content increased by 4.3-fold to 17.1-fold compared with the receptor material, which is a very large increase when compared with results from other studies. Because the increase in AsA observed in the *uorf_GhGGP1_* mutants exceeded that reported in previous uORF-editing studies, we independently verified this result using UHPLC-QE HRMS. The consistency between the high-resolution mass spectrometric analysis and the initial spectrophotometric assay indicates that the remarkable accumulation of AsA is unlikely to result from analytical interference by other reducing metabolites. Instead, these findings support the conclusion that disruption of the uORF_GhGGP1_ substantially enhances AsA biosynthesis in cotton.

TFs are a group of regulators that play crucial roles in many biological and developmental processes in plants by regulating spatiotemporal gene expression through recognition of speciﬁc DNA sequences in promoters (Mitsuda and Ohme-Takagi 2009). Although the biosynthesis of AsA is most responsive to GGP1 activity in cotton, knowledge about the mechanisms involved in the regulation of *GhGGP1* transcription is fairly limited. TFs *SlHZ24* (Hu et al. 2016) and *SlNFYA10* (Chen et al. 2020) bind to VTC1, GGP1, and GME to promote or reduce AsA synthesis. At present, there is still no research in cotton to find transcription factors that directly regulate the expression of AsA synthesis genes. In this study, we identiﬁed an *APETALA2/ethylene-responsive element binding factor* (AP2/ERF) TF whose expression correlates with *GhGGP1* expression and AsA content, with overexpression leading to larger increases in AsA. Past studies have found that BBM plays a role in regulating early embryo and endosperm development (Li et al. 2019; Chen et al. 2022). These ﬁndings suggest that a variety of BBM sequences in different plant species from the same clade are involved in various metabolic mechanisms, and thus a new function in regulating AsA biosynthesis is presented in this study. Overexpression of *GhBBM* signiﬁcantly enhanced the expression of *GhGGP1* and the content of AsA (Figure S11b and c), and these levels were reduced when *GhBBM* was mutated via CRISPR/Cas9 (Figure S11f and g). Yeast one-hybrid, dual-LUC, and EMSA showed that *GhBBM* acts by binding the uORF of *GhGGP1* and activating *GhGGP1* transcription (Fig. 4). It is noteworthy that we identified binding sites for transcription factors on the uORF that also interact with the mORF. The expression of *GhGGP1* is not only transcriptionally regulated but also regulated at the translational level through the uORF in the 5'UTR.

Although cotton has a certain drought tolerance, long-term drought will have a significant impact on its growth and development, plant type structure, number of bolls, and fiber quality (Gupta et al. 2020). Under drought stress or water deficit, cotton not only shows morphological changes in leaves and roots but also regulates its internal physiological and biochemical substances. These include changes in substances involved in osmotic regulation, ROS scavengers, endogenous hormones, and other substances, which jointly cope with the challenges brought by drought stress (Upadhyaya et al. 2011; Choudhury et al. 2017; Agurla et al. 2018; Gupta et al. 2020). AsA is the most abundant antioxidant in plants, which can help plants remove excess ROS, protect plants from oxidative damage, and improve plant stress resistance (Macknight et al. 2017; Edwards et al. 2022). *AtERF98* regulates AsA biosynthesis by directly regulating the expression of AsA synthesis gene VTC1, thereby enhancing salt tolerance in Arabidopsis (Zhang et al. 2012). *NtRAV4* negatively regulates plant tolerance to drought stress by reducing water loss and activating the antioxidant system and stress-related gene expression to maintain the steady state of the ROS system (Gao et al. 2022). Protein tyrosine phosphatase nucleotidase is a key regulatory node that mediates crosstalk between ABA signaling and AsA biosynthesis, thereby promoting plant drought resistance (Zhang et al. 2020). In this study, we found for the first time that *GhBBM* directly regulates the expression of AsA synthesis gene *GhGGP1* in cotton and enhances the drought tolerance of cotton. Therefore, both could be potentially useful new tools for biotech manipulation targeting increased plant resiliency to abiotic stresses. We have experimentally verified the regulatory role of *GhBBM* on *GhGGP1* mRNA in vivo through qRT-PCR analyses. This finding raises the intriguing possibility that *GhBBM* exerts its effect by disrupting uORF-mediated translational repression or by modulating promoter element activity. In theory, this could be beneficial under stress conditions when AsA is required for response to stress such as drought, for instance. Further work is required to verify *mRNA* binding as well as *GhBBM* expression patterns under stress conditions.

An additional observation is that the binding motif is only present in *GhGGP1-A01* and *GhGGP1-D01*, suggesting that the expression of *GhGGP1-A13*, *GhGGP1-D13*, and *GhGGP1-D05b* would not be stimulated by GhBBM. How this might influence drought tolerance in the cotton germplasm would be another avenue to explore, as well as whether germplasm exists that has alleles that do have this binding motif.

Generally, a higher level of crop resistance will lead to a decrease in yield, a phenomenon known as the “growth defense tradeoff,” involving energy and resource allocation, hormone regulation, gene expression regulation, and other aspects (Liu et al. 2019; Yin et al. 2024). In this study, the *uorf_GhGGP1_*, OE-*GhBBM*, and KO-*GhBBM* transgenic materials we created maintained stable fiber quality and yield under normal conditions, achieving a balance between yield and resistance (Table S2). Our study provides a theoretical basis and practical guidance for cultivating high-yield and high-resistance crop varieties.

Although *GhBBM* expression was detected predominantly in stems (Figure S9), we measured drought-related physiological parameters in leaves because these measurements reflect systemic responses of the whole plant to drought. Our stem-dye tracing experiments demonstrated that *GhBBM* affects stem water transport under drought conditions, and transgenic lines with enhanced stem transport capacity also exhibited significantly higher relative water content (Figure S12). These findings support a model in which stem-localized *GhBBM* enhances whole-plant drought tolerance by improving stem transport function and maintaining water homeostasis. Thus, the observed drought-tolerance phenotypes and physiological changes in leaves are likely secondary consequences of improved systemic water homeostasis rather than a direct action of *GhBBM* in leaf cells.

In summary, our findings demonstrate that GhBBM directly binds to a cis-acting element within the uORF_GhGGP1_ and activates its expression. Editing of the uORF_GhGGP1_ promotes GGP1 translation, elevates its protein abundance, and consequently increases AsA content, thereby enhancing drought tolerance in cotton plants (Fig. 6). Our results delineate how the uORF_GhGGP1_ mediates drought resistance via a dual regulatory mechanism. This work not only reveals a multi-layered regulatory cascade integrating transcriptional and translational control but also highlights the potential of uORF editing to optimize crop stress resistance without interfering with native promoter architecture.

**Figure 6:**
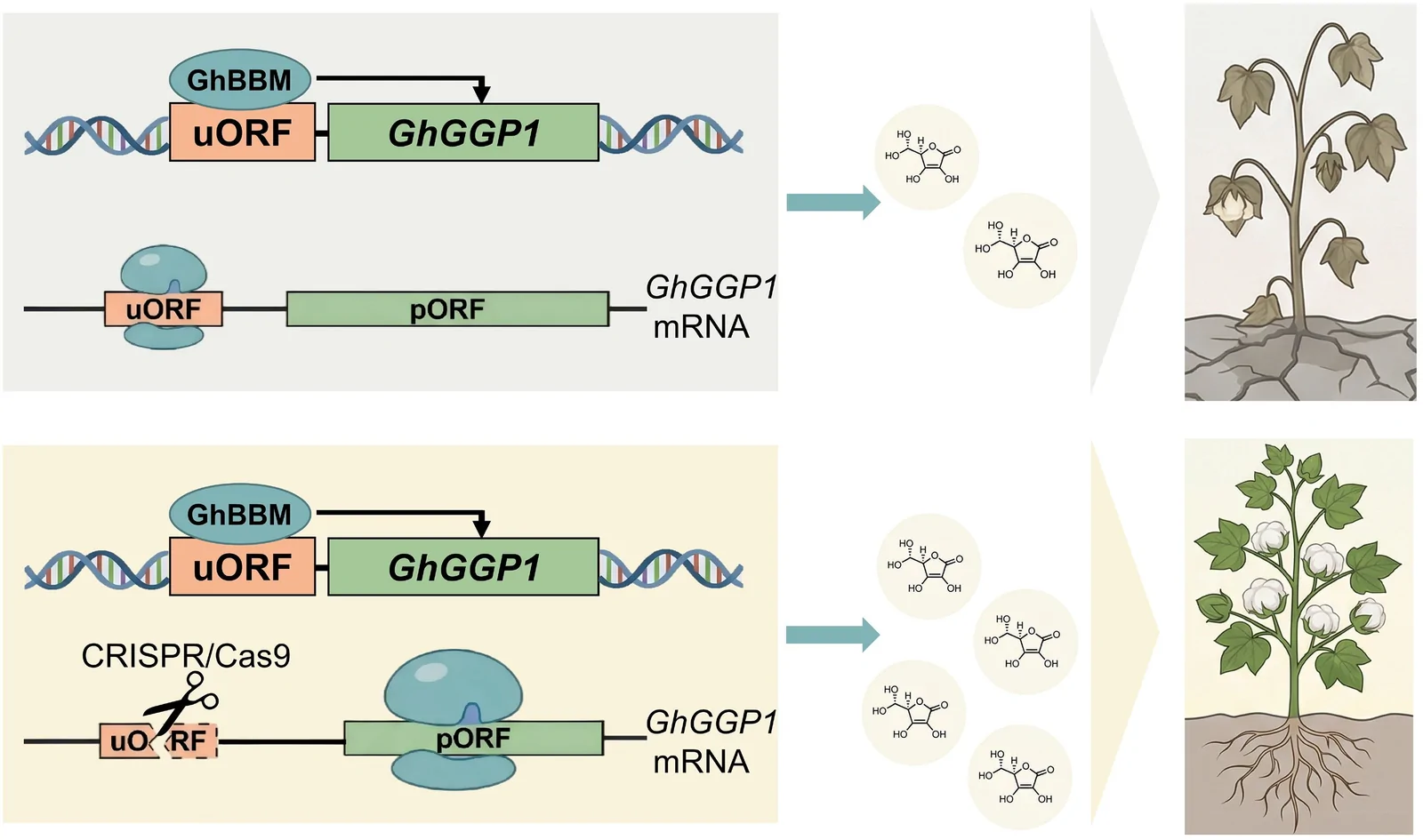
Dual-layer regulation of *GhGGP1* via its uORF enhances drought tolerance in cotton. GhBBM directly binds to a cis-regulatory element within the uORF_GhGGP1_, positively activating its transcription and promoting AsA accumulation. Under normal conditions, the uORF suppresses translation of the pORF by limiting ribosomal access. CRISPR/Cas9-mediated disruption of this uORF relieves translational repression without altering the coding sequence, thereby increasing GGP1 protein abundance. Elevated GGP1 enhances AsA biosynthesis and antioxidant capacity, ultimately improving drought tolerance in cotton. Collectively, this model underscores uORF editing as a precise regulatory strategy that integrates transcriptional activation with translational control to optimize crop stress adaptation.

## Supplementary Material

kiag693_Supplementary_Data

## Data Availability

All data supporting the findings of this study are available within the article and its supplementary information files.
