# Supplementary material for: An investigation of the dual mechanism of uORF-mediated regulation of GGP1 expression and the effects on drought stress in cotton
Source: Plant Physiol. 2026 Oct 19;202(2):kiag693. doi: 10.1093/plphys/kiag693 (PMC13677794; doi:10.1093/plphys/kiag693)
Supplement: Supplementary file 1 [file kiag693_supplementary_data.zip › Revised_Supplemental_Figures.docx]

**Supporting information**

Figure S1 Amino acid sequence alignment and conserved domain analysis of GGP1 proteins from a range of species.

Figure S2 Sequence alignment and tissue expression patterns of *GhGGP1*.

Figure S3 The 5’-leader sequence of *GhGGP1-A01*.

Figure S4 Schematic maps of the sgRNA targeted sites on the uORF region of *GhGGP1*.

Figure S5 Verification of the T0 cotton uORF mutants.

Figure S6 PCR results of T1 generation transgene-free uORF mutants with Cas9 primer based on the pKSE401 vector.

Figure S7 The comparison of growth between INVSc1 (pYES2-*GhGGP1*) and INVSc1 (pYES2) under normal and drought stress conditions.

Figure S8 Identification of the drought resistance for the *uorf_GhGGP1_* lines in the field.

Figure S9 Heatmap of expression patterns of GhBBM genes in various tissues of cotton.

Figure S10 Creation of OE-*GhBBM* and KO-*GhBBM* materials.

Figure S11 Identification of OE-*GhBBM* and KO-*GhBBM* materials.

Figure S12 Dye tracing and relative water content (RWC) measurements in cotton.

Figure S13 AsA content of WT control, *uorf_GhGGP1_-1*, *uorf_GhGGP1_-2*, *uorf_GhGGP1_-3* and *uorf_GhGGP1_-4* (Violet ultraviolet spectrophotometry).

Table S1 List of the primers used in this study.

Table S2 Partial agronomic traits in ZM24, *uorf_GhGGP1_*, OE-*GhBBM* and KO-*GhBBM*.

Figure S1 Amino acid sequence alignment and conserved domain analysis of GGP1 proteins from a range of species. The HIT motif (HφHφQ, where φ is a hydrophobic amino acid) of the HIT protein superfamily and the KKRP NLS are outlined in black. Ta: *Triticum aestivum*, Hv: *Hordeum vulgare*, Bd: *Brachypodium distachyon*, Os: *Oryza sativa*, Ph: *Panicum hallii*, Zm: *Zea mays*, Gh: *Gossypium hirsutum*, At: *Arabidopsis thaliana*, Sl: *Solanum lycopersicum*, Na: *Nicotiana attenuata*.

Figure S2 Sequence alignment and tissue expression patterns of *GhGGP1*.

(a) Amino acid sequence alignment of *GhGGP1* and *AtVTC2* proteins. Black, rose red, blue, and white background color represents 100%, 80 to 100%, 60 to 80%, and less than 60% conservation of amino acids between proteins, respectively.

(b) Heatmap of expression patterns of seven *GhGGP1* genes in various tissues of cotton.

Figure S3 The 5’-leader sequence of *GhGGP1-A01*. In each synthesized fragment, the cleavage sites of the restriction enzymes KpnI and SalI are highlighted in green (bold) and orange (bold), respectively. The uORFs are colored in red. The initiation codon of uORFs is highlighted in bold red.

Figure S4 Schematic maps of the sgRNA targeted sites on the uORF region of *GhGGP1*. Red bases, initiation codon of the uORF_GhGGP1_; black bold-faced bases, PAM motifs (NGG).

Figure S5 Verification of the T0 cotton uORF mutants.

(a) Detection of Cas9 protein in Z102 and Z103 transformed plants. The tissue culture transformant obtained by infecting and transforming Agrobacterium carrying the pKSE401-uORF_GhGGP1_-A01D05b vector is numbered Z102. Similarly, the tissue culture carrying the pKSE401-uORF_GhGGP1_-D01D13 vector is numbered Z103.

(b) Hi-TOM sequencing results of Z102 transformation. The uORF sequence (blue) is shown with the sgRNA target site underlined and the protospacer-adjacent motif shown in bold. The nucleotide insertion or nucleotide deletion in the mutants is labeled in

red. The putative initiation codon of uORF_GhGGP1_ is boxed.

Figure S6 PCR results of T1 generation transgene-free uORF mutants with Cas9 primer based on the pKSE401 vector. Lanes with no bands generated by the Cas9 primer indicate transgene-free mutants.

Figure S7 The comparison of growth between INVSc1 (pYES2-*GhGGP1*) and INVSc1 (pYES2) under normal and drought stress conditions. The CDS sequence of seven copies of the *GhGGP1* gene was inserted into the yeast expression vector pYES2 to construct a recombinant plasmid, which was then transferred into the sensitive yeast strain INVSc1 to obtain the recombinant yeast. At the same time, the pYES2 vector without the inserted gene was also transferred into INVSc1 as a negative control. Dilute the recombinant yeast and control yeast in concentration gradients of 10^-1^, 10^-2^, 10^-3^, and 10^-4^, respectively, and drop them onto SD/- Ura solid medium containing 0 M, 1 M, and 2 M mannitol, and grow at 28℃ for 3 days. The larger the dilution factor, the more obvious the difference. The image only shows the results of 10^-3^ and 10^-4^ dilution factors.

Figure S8 Identification of the drought resistance for the *uorf_GhGGP1_* lines in the field.

(a) Relative survival rates of the WT control and *uorf_GhGGP1_* lines after using the 3% repeated drought method during the seedling stage. All values represent means ± s.d. *****P* < 0.0001 by One-way ANOVA.

(b) Phenotypes of the WT and the *uorf_GhGGP1_-2* line in the field.

Figure S9 Heatmap of expression patterns of *GhBBM* genes in various tissues of cotton.

Figure S10 Creation of OE-*GhBBM* and KO-*GhBBM* materials.

(a) PCR results of T1 generation transgene OE-*GhBBM*. PCR results of T1 generation transgene-free uORF mutants with Cas9 primer based on the WMC016 vector. Lanes with no bands generated by the Cas9 primer indicate transgene-free mutants.

(b) Comparison of *GhBBM* protein levels among WT control, OE-*GhBBM*-1, and OE-*GhBBM*-2. Actin was used as a loading control in the immunoblot assay. The Flag tag is used on the OE-*GhBBM* vector.

Figure S11. Identification of OE-*GhBBM* and KO-*GhBBM* materials.

(a-b) qRT-PCR analysis of (a) *GhBBM* and (b) *GhGGP1* in wild-type (WT) and three Overexpression-*GhBBM* transgenic cotton (*Gossypium hirsutum* L.) lines. OE-*GhBBM*-1 and OE-*GhBBM*-2 represent two different transgenic lines, respectively.

(c) AsA content of overexpression-*GhBBM* transgenic cotton lines.

(d) Schematic map of the sgRNA targeted sites on the genomic regions of *GhBBM*. Lines, intergenic region and introns; grey boxes, exons; and red bases, PAM motifs (NGG).

(e) Sequencing results and amino acid variation of the *GhBBM* homozygous mutant lines from the T1 generation. Black boldfaced bases, PAM motif; red amino acid , amino acid following bases mutation; blue underlining, three nucleotides correspond to one amino acid; red box, regions where base changes occur; dashes, deletions; and blue letters, insertions, and substitutions.

(f) qRT-PCR analysis of *GhGGP1* in wild-type (WT) and two KO-*GhBBM* transgenic lines.

(g) AsA content of KO-*GhBBM* transgenic cotton lines.

Note that each experiment of (a–c) and (f–g) was performed in three replicates (n > 10). All of the above error bars denote standard deviation (±SD). Significant differences to WT were detected by One-way ANOVA using GraphPad Prism 8: **P* < 0.05; ***P* < 0.01; ****P* < 0.001. All data in this figure are derived from the stems of the corresponding materials.

Figure S12 Dye tracing and relative water content (RWC) measurements in cotton.

(a-b) Acid fuchsin staining tracing test was used to evaluate water transport in the stem. The dye moved upward through the stem. Under normal watering conditions, WT, OE-*GhBBM*, and KO-*GhBBM* exhibited comparable dye migration lengths. After PEG6000 drought treatment, the dye migration distance of OE-*GhBBM* was greater than that of WT, while KO-BBM displayed restricted transport (a). Compared to WT, the stem of the *uorf_GhGGP1_* mutant had stronger water transport capacity (b). Bar = 0.5cm.

(c-d) Quantification of relative water content (RWC) after 10% PEG6000 drought treatment. (c) RWC values of WT, OE-*GhBBM*, and KO-*GhBBM*; (d) RWC comparison between WT and *uorf_GhGGP1_* mutants.

Figure S13 AsA content of WT control, *uorf_GhGGP1_-1*, *uorf_GhGGP1_-2*, *uorf_GhGGP1_-3* and *uorf_GhGGP1_-4* (Violet ultraviolet spectrophotometry).
